# The impact of depression and anxiety disorders on postoperative outcomes for patients having total hip or knee arthroplasty: Protocol of a meta-analytic study from cohort studies

**DOI:** 10.1371/journal.pone.0318067

**Published:** 2025-01-27

**Authors:** Liang Lin, Qing Zhang, Min Xu, Zhihong Xiao, Guosong Xu, Zubing Mei

**Affiliations:** 1 Department of Orthopaedics, The First Hospital of Putian City, The School of Clinical Medicine, Fujian Medical University, Putian, Fujian, China; 2 Department of Orthopedics, Changhai Hospital, Naval Medical University, Shanghai, China; 3 Department of Spine Surgery, Lishui People’s Hospital, The Sixth Affiliated Hospital of Wenzhou Medical University, Lishui, China; 4 Department of Anorectal Surgery, Shuguang Hospital Affiliated to Shanghai University of Traditional Chinese Medicine, Shanghai, China; 5 Anorectal Disease Institute of Shuguang Hospital, Shanghai, China; Monash University Faculty of Medicine Nursing and Health Sciences, AUSTRALIA

## Abstract

**Background and purpose:**

Total hip arthroplasty (THA) and total knee arthroplasty (TKA) are widely performed surgeries for end-stage joint disease, yet the influence of depression and anxiety on postoperative outcomes remains unclear. This study aims to consolidate current evidence on the relationship between preoperative depression and/or anxiety disorders and postoperative outcomes in adult patients undergoing primary THA or TKA. Given the potential for these psychiatric conditions to affect recovery, pain management, and overall satisfaction, the results of this study are crucial to inform targeted perioperative interventions and improve patient-centered care.

**Methods and analysis:**

We will search PubMed, Embase, Cochrane Library and PsycINFO from inception to the November 2024, adopting a comprehensive search strategy with no language restrictions. Eligible studies will include cohort studies evaluating adults with a diagnosis of depression and/or anxiety before THA or TKA compared to those without such disorders. Inclusion criteria will focus on preoperative psychiatric diagnoses, clearly defined postoperative outcomes (such as complications, functional recovery measures, pain, length of stay, and patient-reported outcomes). Risk of bias assessment will be performed using the Newcastle-Ottawa Scale. Meta-analysis will be conducted using a random-effects model to calculate pooled risk estimates and 95% confidence intervals for each outcome. Heterogeneity will be quantified with the I^2^ statistic, and a threshold of I^2^ > 50% will indicate substantial heterogeneity. Sources of heterogeneity will be explored via subgroup analyses or meta-regression if possible. Potential publication bias will be visually assessed using funnel plots and statistically tested using Egger’s test. Sensitivity analyses will be carried out to evaluate the robustness of the results for each outcome through leave-one-out procedure.

**Discussion:**

This study will introduce a systematic and rigorous approach to synthesizing evidence from multiple cohorts, providing a comprehensive understanding of the impact of depression and anxiety on THA and TKA outcomes. The findings will guide clinicians in recognizing and managing mental health issues to optimize postoperative recovery and ultimately improve patient satisfaction and quality of life.

**Study registration:**

**Study registration:** CRD42024500008.

## Introduction

Joint replacement surgeries, particularly total hip arthroplasty (THA) and total knee arthroplasty (TKA), stand as cornerstones in the management of advanced osteoarthritis and other debilitating joint diseases, offering significant pain relief and functional restoration to millions of patients worldwide [1–3]. Despite the proven effectiveness of these procedures, patient outcomes can vary significantly, with factors such as age, comorbidities, and psychological well-being influencing postoperative recovery [4–8]. Among these, depression and anxiety disorders, prevalent among the adult population, have garnered increasing attention due to their potential to affect surgical outcomes [9–16].

Depression and anxiety are known to modulate pain perception, adherence to rehabilitation regimens, and overall patient satisfaction, thereby impacting the success of surgical interventions [16–23]. Patients with these conditions might experience heightened postoperative pain, prolonged rehabilitation periods, and increased risk of complications, potentially leading to suboptimal functional outcomes and reduced quality of life [22, 24–29]. However, the precise magnitude of this effect and its underlying mechanisms remain poorly elucidated in the context of THA and TKA.

Existing literature examining the relationship between preoperative mental health status and surgical outcomes for joint arthroplasty presents a fragmented picture, with studies yielding inconsistent findings. Some report negative associations between depression and anxiety with postoperative functional recovery and patient-reported outcomes [9, 10, 12–15, 30, 31], while others fail to demonstrate a clear correlation [32–34]. This variability may stem from methodological differences, sample heterogeneity, and variations in outcome measures across studies.

Given the critical importance of identifying and addressing psychological barriers to successful rehabilitation, a comprehensive synthesis of available evidence from observational studies is warranted. Such an endeavor would not only clarify the extent to which depression and anxiety disorders influence THA and TKA outcomes but also identify potential areas where targeted interventions could be most beneficial. Observational studies, by capturing data from routine clinical settings, offer direct insights into the real-world impacts of these psychological conditions on surgical outcomes.

Our proposed pooled analysis of cohort studies aims to systematically review and analyze the existing body of evidence on the impact of depression and anxiety disorders on various postoperative outcomes following THA and TKA. Outcomes of interest include, but are not limited to, surgical complications, functional recovery metrics, pain scores, length of hospital stay, and patient-reported satisfaction. Through rigorous methodology, including a comprehensive literature search, meticulous data extraction, and thorough risk of bias assessment, we aspire to deliver a robust and clinically meaningful synthesis of the available data.

Ultimately, this study aims to bridge the gap in knowledge surrounding the interaction between mental health and surgical outcomes in THA and TKA patients. Its findings will inform clinical practice, emphasizing the importance of preoperative mental health screening and intervention strategies, thereby guiding healthcare providers towards more patient-centered, holistic care approaches. By illuminating the role of depression and anxiety in postoperative recovery, this work paves the way for targeted interventions aimed at optimizing surgical outcomes and enhancing the well-being of patients undergoing joint replacement surgeries.

## Methods and analysis

### Protocol and registration

This study protocol will adhere to the Preferred Reporting Items for Systematic Reviews and Meta-Analyses Protocols (PRISMA-P) guidelines [35] (S1 Table). It has been registered within the International Prospective Register of Systematic Reviews (PROSPERO) under the registration number CRD42024500008.

### Population

Our population of interest encompasses adult patients who have undergone primary THA or TKA for end-stage joint disease. Participants must have a documented preoperative diagnosis of depression, anxiety, or both, as well as a comparator group without these psychological conditions. No restrictions will be placed on demographic characteristics such as age, sex, or ethnicity to maintain a broad representation.

### Exposure assessed

The primary exposure of interest is the presence of preoperative depression and/or anxiety disorders, diagnosed according to established clinical criteria. Studies that utilize validated scales or diagnostic interviews to ascertain these conditions will be included, ensuring a standardized assessment of the exposure across the included cohort studies.

### Control population

The control population comprises patients undergoing THA or TKA without a documented diagnosis of depression or anxiety. This group serves as a comparative reference for evaluating the differential impact of these psychological factors on postoperative outcomes.

### Outcomes

The outcomes of interest encompass a spectrum of postoperative results, including risk of surgical complications, revision and readmission, functional recovery outcomes, length of hospital stay, and patient-reported outcomes (such as satisfaction, quality of life, and return to daily activities).

### Eligibility criteria

Publications meeting the following criteria will be included: cohort studies investigating adults undergoing primary THA or TKA, with a clear differentiation between patients diagnosed with depression and/or anxiety preoperatively and those without such diagnoses. Studies must provide a minimum of 6–12 months’ follow-up data on postoperative outcomes. Non-peer-reviewed literature, case reports, reviews, conference abstracts, and studies with insufficient data for analysis will be excluded.

### Search strategy

A systematic search of electronic databases, including PubMed, Embase, the Cochrane Library, and PsycINFO will be conducted from their inception to November 2024. Search terms will include a combination of keywords and Medical Subject Headings (MeSH) related to "total hip arthroplasty," "total knee arthroplasty," "depression," "anxiety disorders," "postoperative outcomes," and "cohort studies." The search strategy will be adapted for each database, and no language restrictions will initially be applied to maximize sensitivity (details see **Tables 1–3**). Reference lists of included studies and relevant reviews will be manually screened to identify additional eligible studies.

**Table 1:** Search strategy for Pubmed.

*Depression or Anxiety terms*
1 "Anxiety"[Mesh] OR "Anxiety Disorders"[Mesh] 2 "Depressive Disorder"[Mesh] OR "Depression"[Mesh] 3 (depress* or dysthymi* or adjustment disorder* or mood disorder* or affective disorder* or affective symptom* or anxiety or anxious or ?phobi* or panic disorder or BPSD or behavioural and psychological symptoms of dementia or neuropsychiatric symptom* or NPS or agoraphobi* or anxio* or panic or obsessi* or compulsi* or OCD or GAD or PTSD or posttrauma* or post-trauma* or post trauma* or stress disorder or neurosis or neuroses or neurotic or psychoneuro*) [Title/Abstract] 4 1-3/or ***Hip or knee replacement terms*:** 5 "Hip Prosthesis"[Mesh] OR "Arthroplasty, Replacement, Hip"[Mesh] 6. "Knee Prosthesis"[Mesh] OR "Arthroplasty, Replacement, Knee"[Mesh] 7. (hip* or knee) [Title/Abstract] 8. (arthroplast* OR prosthe* OR replac* or operat* or surg*) [Title/Abstract] 9. 7 and 8 10. 5 or 6 or 9 ***Study design terms*:** 11 "Retrospective Studies"[Mesh] 12 "Cohort Studies"[Mesh] 13 "Longitudinal Studies"[Mesh] 14 "Follow-Up Studies"[Mesh] 15 "Prospective Studies"[Mesh] 16 "Registries"[Mesh] 17 (cohort or longitudinal or followup or prospective* or retrospective* or database* or population* or follow up or registry or registries or incidence OR prevalence OR mortality OR outcome OR progression OR natural history OR prognos* OR course* OR predict* OR population based OR epidemiologic OR case control) [Title/Abstract] 18 11-17/or ***Final search results*: *Combining Depression or Depressive disorder*, *Risk Factor*, *Suicide and Study design*:** 19 4 and 10 and 18 (1,934)

**Table 2:** Search strategy for Embase.

Depression or Anxiety terms
1 ’anxiety’/exp OR ‘Anxiety Disorders’/exp 2 ’depression’/exp 3 (depress* or dysthymi* or ‘adjustment disorder*’ or ‘mood disorder*’ or ‘affective disorder*’ or ‘affective symptom*’ or anxiety or anxious or ?phobi* or ‘panic disorder’ or BPSD or ‘behavioural and psychological symptoms of dementia’ or ‘neuropsychiatric symptom*’ or NPS or agoraphobi* or anxio* or panic or obsessi* or compulsi* or OCD or GAD or PTSD or posttrauma* or post-trauma* or ‘post trauma*’ or ‘stress disorder’ or neurosis or neuroses or neurotic or psychoneuro*):ab,ti 4 1-3/or **Hip or knee replacement terms:** 5 ’hip prosthesis’/exp OR ’hip replacement’/exp 6. ’knee prosthesis’/exp OR ’knee replacement’/exp 7. (hip* or knee):ab,ti 8. (arthroplast* OR prosthe* OR replac* or operat* or surg*):ab,ti 9. 7 and 8 10. 5 or 6 or 9 **Study design terms:** 11 ’retrospective study’/exp 12 ’cohort analysis’/exp 13 ’longitudinal study’/exp 14 ’follow up’/exp 15 ’prospective study’/exp 16 ’register’/exp 17 (cohort or longitudinal or followup or prospective* or retrospective* or database* or population* or ‘follow up’ or registry or registries or incidence OR prevalence OR mortality OR outcome OR progression OR ‘natural history’ OR prognos* OR course* OR predict* OR ‘population based’ OR epidemiologic OR ‘case control’):ab,ti 18 11-17/or **Final search results: Combining Depression or Depressive disorder, Risk Factor, Suicide and Study design:** 18 4 and 10 and 18

**Table 3:** Search strategy for Cochrane Library.

*Depression or Anxiet terms*
1 MeSH descriptor: [Anxiety] explode all trees OR MeSH descriptor: [Anxiety Disorders] explode all trees 2 MeSH descriptor: [Depressive Disorder] explode all trees OR MeSH descriptor: [Depression] explode all trees 3 (depress* or dysthymi* or adjustment disorder* or mood disorder* or affective disorder* or affective symptom* or anxiety or anxious or ?phobi* or panic disorder or BPSD or behavioural and psychological symptoms of dementia or neuropsychiatric symptom* or NPS or agoraphobi* or anxio* or panic or obsessi* or compulsi* or OCD or GAD or PTSD or posttrauma* or post-trauma* or post trauma* or stress disorder or neurosis or neuroses or neurotic or psychoneuro*):ti,ab,kw 4 1-3/or ***Hip or knee replacement terms*:** 5 MeSH descriptor: [Hip Prosthesis] explode all trees OR MeSH descriptor: [Arthroplasty, Replacement, Hip] explode all trees 6. MeSH descriptor: [Knee Prosthesis] explode all trees OR MeSH descriptor: [Arthroplasty, Replacement, Knee] explode all trees 7. (hip* or knee):ti,ab,kw 8. (arthroplast* OR prosthe* OR replac* or operat* or surg*):ti,ab,kw 9. 7 and 8 10. 5 or 6 or 9 ***Study design terms*:** 11 MeSH descriptor: [Retrospective Studies] explode all trees 12 MeSH descriptor: [Cohort Studies] explode all trees 13 MeSH descriptor: [Longitudinal Studies] explode all trees 14 MeSH descriptor: [Follow-Up Studies] explode all trees 15 MeSH descriptor: [Prospective Studies] explode all trees 16 MeSH descriptor: [Registries] explode all trees 17 (cohort or longitudinal or followup or prospective* or retrospective* or database* or population* or follow up or registry or registries or incidence OR prevalence OR mortality OR outcome OR progression OR natural history OR prognos* OR course* OR predict* OR population based OR epidemiologic OR case control):ti,ab,kw 18 11-17/or ***Final search results*: *Combining Depression or Depressive disorder*, *Risk Factor*, *Suicide and Study design*:** 19 4 and 10 and 18

### Selection process

Identified records will be managed using EndNote reference management software. Titles and abstracts will be screened independently by two reviewers to determine relevance. Studies meeting the inclusion criteria or those with insufficient information for exclusion will proceed to full-text review. Full-text assessments will also be performed independently by the two reviewers, with any disagreements resolved through discussion or consultation with a third reviewer. Reasons for exclusion at both stages will be documented.

### Data collection

Data extraction will be executed by two independent reviewers using a predefined, piloted data extraction form. Extracted data will encompass study characteristics (author, year, country), participant demographics, details of the exposure (diagnostic criteria for depression/anxiety), intervention specifics, comparators, and all predefined outcomes of interest. Where feasible, missing data will be requested from study authors. Data extraction process will be cross-checked for accuracy and consistency, with any discrepancies addressed through consensus.

### Risk of bias assessment

To ensure the reliability and validity of the synthesized evidence, a rigorous risk of bias assessment will be conducted for all included studies. For non-randomized cohort studies, the ROBINS-I tool will be applied to evaluate seven domains of bias: confounding, selection of participants into the study, classification of interventions, deviations from intended interventions, missing data, measurement of outcomes, and selection of reported results [36]. Judgments for each domain will inform an overall risk of bias assessment for each study, categorized as ’low’, ’moderate’, ’serious’, or ’critical’. Disagreements in the risk of bias assessment will also be resolved through discussion among reviewers or consultation with a third expert reviewer when necessary.

### Quality of evidence

The quality of evidence for each outcome will be assessed using the Grading of Recommendations, Assessment, Development, and Evaluation (GRADE) approach [37]. This system categorizes evidence into four levels: high, moderate, low, and very low, based on study design, consistency, directness, precision, publication bias, and other factors influencing the confidence in the estimation of the effect magnitude.

Given the nature of our study, which primarily includes cohort studies, the initial grade for the quality of evidence will start as ’low’ due to the inherent limitations of observational designs compared to randomized controlled trials. However, we acknowledge the potential for upgrading the quality of evidence in certain circumstances. Upgrading may occur if there is a large magnitude of effect observed consistently across studies, implying a strong and consistent association between depression/anxiety and postoperative outcomes in THA and TKA patients. Additionally, evidence could be upgraded if there is evidence of a dose-response relationship, suggesting a biological gradient that strengthens the causal inference.

On the other hand, downgrading of evidence may take place if there is substantial unexplained heterogeneity among study results, suggesting inconsistency across the body of evidence. Furthermore, imprecision due to wide confidence intervals or small sample sizes, indirectness if the population, intervention, comparator, or outcomes do not align precisely with our research question, or high risk of bias as assessed by the NOS, can also lead to a lower quality rating.

### Data analysis

Data synthesis will involve a random-effects meta-analysis to account for anticipated heterogeneity between studies [38]. Pooled risk ratios (RR) with corresponding 95% confidence intervals (CI) will be calculated for dichotomous outcomes, such as complication rates or achievement of specific functional milestones. For continuous outcomes like functional recovery measures, weighted mean differences (WMD) or standardized mean differences (SMD) with 95% CI will be estimated. Heterogeneity among studies will be quantified using the I^2^ statistic, with values above 50% indicating substantial heterogeneity [39]. Subgroup analyses will be conducted based on factors such as age group (adult vs. elderly), type of arthroplasty (THA vs. TKA), severity of depression/anxiety, severity of preoperative pain, and study quality, to explore potential sources of heterogeneity. Publication bias will be assessed visually with funnel plots and statistically tested using Egger’s test [40]. If substantial heterogeneity is detected, meta-regression will be considered to investigate the influence of specific study-level characteristics on the effect sizes. Sensitivity analyses will be carried out to test the robustness of our findings against potential outliers or methodological variations. All statistical analyses will be performed using STATA 12.0 software, and a p value less than 0.05 will be considered statistical significance.

### Patient and public involvement

Patients and the public were not directly involved in the development of this study’s design, execution, or the drafting of this protocol. However, the research questions and outcomes were formulated with a view to address concerns pertinent to patients undergoing THA and TKA, aiming to improve their postoperative experiences and overall well-being. Future dissemination plans include summaries tailored for patient audiences to ensure findings are accessible and understandable, encouraging dialogue and empowering informed decisions.

### Ethics and dissemination

As this study involves a synthesis of already published data, ethical approval is not required. Nevertheless, all aspects of the study will be conducted in accordance with ethical research principles. Upon completion, the findings will be disseminated through publication in a reputable, peer-reviewed scientific journal, complemented by presentations at national and international orthopedic and mental health conferences. Additionally, summaries will be shared with relevant patient organizations and healthcare professionals to facilitate implementation of the evidence-based insights into clinical practice.

### Amendments

Any amendments to the protocol during the course of the study, including changes to the methods or outcomes, will be documented with clear justifications. Amendments will be communicated to all team members, registered in the PROSPERO database (CRD42024500008), and reflected in the final publication to ensure transparency and accurate representation of the research process. Adjustments necessitated by new information or methodological considerations will be made with the utmost scrutiny to preserve the integrity and scientific rigor of the study.

## Discussion

### Principal findings

Our systematic review and meta-analysis will compile and critically appraise the existing literature to elucidate the connection between depression and anxiety disorders in patients undergoing THA or TKA and their subsequent postoperative outcomes. By consolidating data from multiple cohort studies, we aim to clarify the extent to which these psychological factors influence surgical recovery, functional restoration, and overall patient satisfaction. The principal findings are expected to offer quantitative evidence on the differential impact of depression and anxiety, potentially revealing a graded relationship with varying severities or types of these disorders.

### Potential mechanisms

Understanding the mechanisms behind the observed relationships is crucial for informing clinical practice. Theoretical frameworks suggest that depression and anxiety may interfere with neuroendocrine and immune responses, exacerbating inflammation and slowing tissue healing post-surgery [41–45]. Moreover, these conditions can negatively affect patient engagement in rehabilitation, adherence to medication regimes, and pain coping strategies, all of which are pivotal to optimal recovery. By synthesizing the available evidence, our study may shed light on these pathways and prompt further investigation into the biological and behavioral mechanisms underlying the observed associations.

### Strengths and limitations

A key strength of our study lies in its systematic and rigorous methodology, following PRISMA-P guidelines, which ensures a comprehensive and unbiased review of the literature. The inclusion of a broad range of outcomes and the application of GRADE criteria to assess the quality of evidence contribute to a robust analysis. However, potential limitations include the inherent biases associated with observational studies, language restrictions, and the possibility of unmeasured confounders in the included studies. The reliance on published data may also introduce publication bias. Nonetheless, sensitivity analyses and subgroup explorations will be conducted to mitigate these limitations and enhance the validity of our conclusions. Furthermore, while this meta-analysis provides comprehensive insights from observational studies, it is important to acknowledge that only observational studies were included. The exclusion of randomized controlled trials, due to their limited availability on the subject, may affect the generalizability of the findings. Observational studies can include biases not typically present in randomized controlled trials, which should be considered when interpreting the results.

### Clinical significance

Our findings hold significant clinical implications. By quantifying the impact of depression and anxiety on arthroplasty outcomes, healthcare providers can better appreciate the need for preoperative mental health screenings and interventions. This may lead to tailored prehabilitation programs integrating psychological support, improving patient preparedness, and potentially reducing postoperative complications and enhancing recovery. Additionally, the identification of high-risk patient groups could facilitate targeted follow-up care and personalized rehabilitation strategies.

## Conclusion

In summary, our systematic review and pooled analysis aim to provide a definitive synthesis of the current knowledge on how depression and anxiety disorders affect THA and TKA outcomes. The study’s results will not only inform clinical decision-making by highlighting the importance of considering psychological factors in perioperative care but also stimulate further research into the development of evidence-based interventions. Ultimately, this work strives to contribute to enhancing patient-centered care and improving the quality of life for individuals undergoing these surgeries.

## Supporting information

S1 Table PRISMA-P checklist. (DOC)
