# Supplementary material for: The impact of depression and anxiety disorders on postoperative outcomes for patients having total hip or knee arthroplasty: Protocol of a meta-analytic study from cohort studies
Source: PLoS One. 2025 Jan 27;20(1):e0318067. doi: 10.1371/journal.pone.0318067 (PMC11771941; doi:10.1371/journal.pone.0318067)
Supplement: Attachment — Submitted filename: Response to Reviewers.docx [file pone.0318067.s002.docx]

**Detailed Rebuttal Letter to Editor and Reviewer**

**Ms. No.: PONE-D-24-55368**

To: The impact of depression and anxiety disorders on postoperative outcomes for patients having total hip or knee arthroplasty: protocol of a meta-analytic study from cohort studies

Prof. Sultana Monira Hussain,

Academic Editor

***PLOS ONE***

Dear Prof. Sultana Monira Hussain,

Thank you for forwarding the review comments to us. We have carefully gone through the feedback and have made the necessary changes to our manuscript. We appreciate the insightful feedback provided by the editor and reviewers, as they have helped us improve the quality of our study. Please find below a detailed list of the changes made in response to the Editor and the Reviewer's concerns:

**Editor’s comments**

Thank you for submitting your manuscript to PLOS ONE. After careful consideration, we feel that it has merit but does not fully meet PLOS ONE’s publication criteria as it currently stands. Therefore, we invite you to submit a revised version of the manuscript that addresses the points raised during the review process.

Please submit your revised manuscript by Jan 29 2025 11:59PM. If you will need more time than this to complete your revisions, please reply to this message or contact the journal office at plosone@plos.org. When you're ready to submit your revision, log on to https://www.editorialmanager.com/pone/ and select the 'Submissions Needing Revision' folder to locate your manuscript file.

Please include the following items when submitting your revised manuscript:

A rebuttal letter that responds to each point raised by the academic editor and reviewer(s). You should upload this letter as a separate file labeled 'Response to Reviewers'.

A marked-up copy of your manuscript that highlights changes made to the original version. You should upload this as a separate file labeled 'Revised Manuscript with Track Changes'.

An unmarked version of your revised paper without tracked changes. You should upload this as a separate file labeled 'Manuscript'.

If you would like to make changes to your financial disclosure, please include your updated statement in your cover letter. Guidelines for resubmitting your figure files are available below the reviewer comments at the end of this letter.

If applicable, we recommend that you deposit your laboratory protocols in protocols.io to enhance the reproducibility of your results. Protocols.io assigns your protocol its own identifier (DOI) so that it can be cited independently in the future. For instructions see: https://journals.plos.org/plosone/s/submission-guidelines#loc-laboratory-protocols. Additionally, PLOS ONE offers an option for publishing peer-reviewed Lab Protocol articles, which describe protocols hosted on protocols.io. Read more information on sharing protocols at https://plos.org/protocols?utm_medium=editorial-email&utm_source=authorletters&utm_campaign=protocols.

We look forward to receiving your revised manuscript.

Kind regards,

Sultana Monira Hussain

Academic Editor

PLOS ONE.

**Response:** Thank you for your consideration of our manuscript. We appreciate the opportunity to revise our manuscript to meet PLOS ONE’s publication criteria and address the review comments comprehensively.

We acknowledge the deadline of January 29, 2025, for the submission of the revised manuscript. We anticipate that we will be able to complete the necessary revisions by this date. However, should we require additional time, we will notify you via [plosone@plos.org](mailto:plosone@plos.org).

In our revised submission, we will include the following documents as instructed:

1. A rebuttal letter addressing each point raised by the academic editor and reviewers, detailing the changes made in response to their feedback.
2. A marked-up copy of the manuscript highlighting the revisions.
3. A clean version of the manuscript without tracked changes for ease of reading.
4. An updated financial disclosure in the cover letter, if applicable.

We also appreciate the suggestion to deposit our laboratory protocols in protocols.io to enhance reproducibility. As this is a study protocol, all the methods have been described in details in the full manuscript.

Thank you for the opportunity to improve our manuscript. We look forward to the possibility of contributing to PLOS ONE and will ensure our revised submission fulfills the journal’s standards for publication.

Journal Requirements:

When submitting your revision, we need you to address these additional requirements.

1. Please ensure that your manuscript meets PLOS ONE's style requirements, including those for file naming. The PLOS ONE style templates can be found at

https://journals.plos.org/plosone/s/file?id=wjVg/PLOSOne_formatting_sample_main_body.pdf and

https://journals.plos.org/plosone/s/file?id=ba62/PLOSOne_formatting_sample_title_authors_affiliations.pdf.

**Response:** Thank you for the guidance regarding the PLOS ONE style requirements. We acknowledge the importance of adhering to the journal's formatting standards and have made the necessary revisions to comply with these guidelines.

Using the PLOS ONE templates provided in the links, we have reformatted our manuscript to match the required style. This includes adjustments to the title page, headings, subheadings, font sizes, and the overall layout to ensure compliance with the journal’s formatting guidelines.

We hope these revisions meet the journal's requirements. Attached are the revised files for your review and consideration. We appreciate the opportunity to refine our manuscript and look forward to the possibility of its acceptance in PLOS ONE.

2. Your ethics statement should only appear in the Methods section of your manuscript. If your ethics statement is written in any section besides the Methods, please move it to the Methods section and delete it from any other section. Please ensure that your ethics statement is included in your manuscript, as the ethics statement entered into the online submission form will not be published alongside your manuscript.

**Response:** Thank you for your guidance regarding the placement of the ethics statement in our manuscript. We understand the importance of correctly positioning this statement to adhere to the journal's standards.

We have carefully reviewed our manuscript and have made the following adjustments:

1. We have moved the ethics statement exclusively to the Methods section of our manuscript. This adjustment ensures that all ethical disclosures are appropriately centralized and easily accessible, aligning with best practices for transparency and reproducibility.
2. We have removed the ethics statement from Abstract section of the manuscript where it previously appeared, to prevent redundancy and maintain a clean and focused presentation of our research.
3. We have ensured that the ethics statement is included in the manuscript as per your instructions. This will guarantee that the statement is available in the published article, as the ethics information provided during the online submission process is not published alongside the manuscript.

We appreciate your attention to the ethical aspects of publication and adherence to PLOS ONE’s policies. We hope these revisions satisfactorily address your comments regarding the ethics statement.

**Additional Editor Comments:**

Dear Authors,

Thank you for your submission. I have a few suggestions that I believe will enhance the rigor and depth of your study:

Bias Assessment: I recommend using a more robust tool for bias assessment, such as the ROBINS-I or JBI tools, rather than the Newcastle-Ottawa Scale. The latter relies on a points system that may not adequately reflect the complexity of bias in observational studies.

**Response:** Thank you for your valuable comments and suggestions aimed at enhancing the rigor of our study. We appreciate your recommendation regarding the bias assessment tool and agree that a more robust method would strengthen our meta-analysis. We acknowledge the limitations of the Newcastle-Ottawa Scale (NOS) in reflecting the complexity of bias in observational studies. In response to your suggestion, we have decided to adopt the ROBINS-I tool for bias assessment, which is specifically designed for reviewing non-randomized studies of interventions. This tool will allow us to more comprehensively assess biases due to confounding, selection of participants, classification of interventions, deviations from intended interventions, missing data, measurement of outcomes, and selection of reported results.

**Revised Method Text:**

"**Risk of Bias Assessment**
To ensure the reliability and validity of the synthesized evidence, a rigorous risk of bias assessment will be conducted for all included studies. For non-randomized cohort studies, the ROBINS-I tool will be applied to evaluate seven domains of bias: confounding, selection of participants into the study, classification of interventions, deviations from intended interventions, missing data, measurement of outcomes, and selection of reported results [37]. Judgments for each domain will inform an overall risk of bias assessment for each study, categorized as 'low', 'moderate', 'serious', or 'critical'. Disagreements in the risk of bias assessment will also be resolved through discussion among reviewers or consultation with a third expert reviewer when necessary.

(Page 13)

Exposure Assessment: For evaluating exposures such as depression and anxiety, I suggest incorporating PsycINFO into your database search strategy. This database specializes in psychological literature and may provide additional relevant studies.

**Response:** Thank you for your constructive suggestion regarding the enhancement of our search strategy. We agree that incorporating PsycINFO is a valuable addition that could significantly enrich our review by providing comprehensive coverage of psychological literature, which is crucial for our research focus on depression and anxiety. We recognize the importance of thoroughly capturing studies related to the psychological aspects of orthopedic postoperative outcomes. PsycINFO, with its specialized focus, will likely yield additional relevant studies that may not be indexed in the databases previously included in our search strategy.

**Revised Abstract Text:**

“**Methods and Analysis:**

We will search PubMed, Embase, Cochrane Library and PsycINFO from inception to the November 2024, adopting a comprehensive search strategy with no language restrictions.”

(Page 4)

**Revised Method Text:**

"**Search Strategy**
A systematic search of electronic databases, including PubMed, Embase, the Cochrane Library, and PsycINFO will be conducted from their inception to November 2024. Search terms will include a combination of keywords and Medical Subject Headings (MeSH) related to "total hip arthroplasty," "total knee arthroplasty," "depression," "anxiety disorders," "postoperative outcomes," and "cohort studies." The search strategy will be adapted for each database, and no language restrictions will initially be applied to maximize sensitivity (details see Table 1-3). Reference lists of included studies and relevant reviews will be manually screened to identify additional eligible studies.”

(Page 11-12)

Subgroup Analysis: In addition to the proposed subgroup analyses, I suggest including a subgroup analysis based on the severity of pain prior to arthroplasty. This variable may influence both psychological factors and postoperative outcomes.

**Response:** Thank you for your insightful comment regarding the incorporation of pain severity in our subgroup analyses. We agree that the severity of preoperative pain is a critical factor that could influence both the psychological state of patients and their outcomes after total hip or knee arthroplasty.

Including a subgroup analysis based on the severity of preoperative pain will allow us to more deeply understand how pain interacts with depression and anxiety to affect postoperative results. Pain severity can significantly affect patient psychology, recovery, and satisfaction with surgical outcomes, thus it is indeed a pertinent variable to include in our analyses.

**Revised Method Text:**

" Subgroup analyses will be conducted based on factors such as age group (adult vs. elderly), type of arthroplasty (THA vs. TKA), severity of depression/anxiety, severity of preoperative pain, and study quality, to explore potential sources of heterogeneity."

(Page 15)

Inclusion of RCTs: If available, I recommend incorporating randomized controlled trials (RCTs) that specifically address the management of psychological disorders (e.g., depression, anxiety) before performing total knee arthroplasty (TKA) or total hip arthroplasty (THA).

**Response:** Thank you for the reviewer's suggestion to include randomized controlled trials (RCTs) that address the management of psychological disorders before total knee arthroplasty (TKA) or total hip arthroplasty (THA). We appreciate the potential value that such studies might offer in understanding the impact of treatment for psychological disorders on surgical outcomes.

However, after careful consideration and a thorough search of the available literature, we have found that RCTs specifically examining the impact of treating depression and anxiety before TKA and THA are exceedingly scarce. The primary reason for this lack of RCTs is the ethical and logistical challenges associated with randomly assigning patients to different treatment modalities for psychological conditions in the context of preparing for major surgery. Such interventions typically require personalized treatment plans that take into account the severity and specifics of the individual's psychological condition, which do not lend themselves well to randomization.

Moreover, the inclusion of RCTs in our study would potentially introduce a significant methodological shift from our original design, which is focused on observational cohort studies. Cohort studies are particularly suitable for our research question as they allow the examination of real-world outcomes in routine clinical practice, providing insights into the naturalistic effects of depression and anxiety on surgical results.

Additionally, the heterogeneity in intervention types, variability in the timing of interventions relative to surgical dates, and differences in outcome measures reported in RCTs compared to observational studies would complicate the synthesis of data and could lead to misleading conclusions.

Therefore, we have chosen to focus our meta-analysis on cohort studies, which provide a more homogeneous and directly applicable body of evidence to assess the impact of pre-existing depression and anxiety on postoperative outcomes in TKA and THA patients.
