# Supplementary material for: The impact of depression and anxiety disorders on postoperative outcomes for patients having total hip or knee arthroplasty: Protocol of a meta-analytic study from cohort studies
Source: PLoS One. 2025 Jan 27;20(1):e0318067. doi: 10.1371/journal.pone.0318067 (PMC11771941; doi:10.1371/journal.pone.0318067)
Supplement: Attachment — Submitted filename: Response to Reviewers.docx [file pone.0318067.s003.docx]

**Detailed Rebuttal Letter to Editor and Reviewer**

**Ms. No.: PONE-D-24-55368R1**

To: The impact of depression and anxiety disorders on postoperative outcomes for patients having total hip or knee arthroplasty: protocol of a meta-analytic study from cohort studies

Prof. Sultana Monira Hussain,

Academic Editor

***PLOS ONE***

Dear Prof. Sultana Monira Hussain,

Thank you for forwarding the review comments to us. We have carefully gone through the feedback and have made the necessary changes to our manuscript. We appreciate the insightful feedback provided by the editor and reviewers, as they have helped us improve the quality of our study. Please find below a detailed list of the changes made in response to the Editor and the Reviewer's concerns:

**Editor’s comments**

Thank you for submitting your manuscript to PLOS ONE. After careful consideration, we feel that it has merit but does not fully meet PLOS ONE’s publication criteria as it currently stands. Therefore, we invite you to submit a revised version of the manuscript that addresses the points raised during the review process.

Please submit your revised manuscript by Feb 20 2025 11:59PM. If you will need more time than this to complete your revisions, please reply to this message or contact the journal office at plosone@plos.org. When you're ready to submit your revision, log on to https://www.editorialmanager.com/pone/ and select the 'Submissions Needing Revision' folder to locate your manuscript file.

Please include the following items when submitting your revised manuscript:

A rebuttal letter that responds to each point raised by the academic editor and reviewer(s). You should upload this letter as a separate file labeled 'Response to Reviewers'.

A marked-up copy of your manuscript that highlights changes made to the original version. You should upload this as a separate file labeled 'Revised Manuscript with Track Changes'.

An unmarked version of your revised paper without tracked changes. You should upload this as a separate file labeled 'Manuscript'.

If you would like to make changes to your financial disclosure, please include your updated statement in your cover letter. Guidelines for resubmitting your figure files are available below the reviewer comments at the end of this letter.

If applicable, we recommend that you deposit your laboratory protocols in protocols.io to enhance the reproducibility of your results. Protocols.io assigns your protocol its own identifier (DOI) so that it can be cited independently in the future. For instructions see: https://journals.plos.org/plosone/s/submission-guidelines#loc-laboratory-protocols. Additionally, PLOS ONE offers an option for publishing peer-reviewed Lab Protocol articles, which describe protocols hosted on protocols.io. Read more information on sharing protocols at https://plos.org/protocols?utm_medium=editorial-email&utm_source=authorletters&utm_campaign=protocols.

We look forward to receiving your revised manuscript.

Kind regards,

Sultana Monira Hussain

Academic Editor

PLOS ONE.

**Response:** Thank you for your consideration of our manuscript. We appreciate the opportunity to revise our manuscript to meet PLOS ONE’s publication criteria and address the review comments comprehensively.

We acknowledge the deadline of January 29, 2025, for the submission of the revised manuscript. We anticipate that we will be able to complete the necessary revisions by this date. However, should we require additional time, we will notify you via [plosone@plos.org](mailto:plosone@plos.org).

In our revised submission, we will include the following documents as instructed:

1. A rebuttal letter addressing each point raised by the academic editor and reviewers, detailing the changes made in response to their feedback.
2. A marked-up copy of the manuscript highlighting the revisions.
3. A clean version of the manuscript without tracked changes for ease of reading.
4. An updated financial disclosure in the cover letter, if applicable.

We also appreciate the suggestion to deposit our laboratory protocols in protocols.io to enhance reproducibility. As this is a study protocol, all the methods have been described in details in the full manuscript.

Thank you for the opportunity to improve our manuscript. We look forward to the possibility of contributing to PLOS ONE and will ensure our revised submission fulfills the journal’s standards for publication.

Journal Requirements:

Please review your reference list to ensure that it is complete and correct. If you have cited papers that have been retracted, please include the rationale for doing so in the manuscript text, or remove these references and replace them with relevant current references. Any changes to the reference list should be mentioned in the rebuttal letter that accompanies your revised manuscript. If you need to cite a retracted article, indicate the article’s retracted status in the References list and also include a citation and full reference for the retraction notice.

**Response:** Thank you for the feedback. We have reviewed our reference list thoroughly to ensure it is complete, accurate, and up-to-date. None of the articles cited in our manuscript have been retracted. We have confirmed this by checking each citation against databases like Retraction Watch and PubMed. Furthermore, all references are now double-checked for accuracy and completeness, adhering to the journal's citation style.

**Additional Editor Comments:**

Thank you for your detailed response and the thorough explanation regarding the inclusion of randomized controlled trials (RCTs). I appreciate the thoughtful rationale you have provided. However, to ensure the clarity and robustness of your manuscript, I recommend addressing the following points in your revision:

At the beginning of the manuscript, please clearly articulate why observational studies were chosen as the primary focus. Highlight their relevance and applicability to your research question. This will help set the stage for readers and provide a clear framework for understanding your approach. Additionally, explicitly acknowledge in the limitations section that you have only included observational studies.

Alternatively, if you decide to include both observational studies and RCTs, please acknowledge the limitations, noting the scarcity of RCTs in this area. Include a brief summary of how many relevant RCTs were identified during your literature search (if any) and the reasons they could not be incorporated. Furthermore, if there are heterogeneities between observational studies and RCTs in your meta-analysis, I suggest conducting a subgroup analysis to account for potential differences.

Addressing these points will enhance the transparency and scientific rigor of your work. I look forward to reviewing your revised manuscript.

Best regards,

Sultana Monira Hussain.

**Response:** Thank you for your detailed comments and suggestions regarding our manuscript. In response to your feedback, we have opted to focus exclusively on observational studies for our meta-analysis. We have made the following revisions to clarify this choice in the manuscript:

In the introduction, we have articulated the rationale for choosing observational studies as our primary focus. This choice was driven by the direct applicability and real-world relevance of these studies to our research question, which investigates the impacts of depression and anxiety on postoperative outcomes in total hip and knee arthroplasty. Observational studies provide valuable insights into patient populations under routine clinical conditions, which is critical for understanding the nuances of how psychological factors affect surgical outcomes.

Furthermore, we have acknowledged in the limitations section that our analysis is based solely on observational studies. This is an important consideration for interpreting the results, as these study designs inherently carry a risk of bias that is typically lower in randomized controlled trials (RCTs). We decided against including RCTs due to their scarcity in examining the psychological aspects of orthopedic outcomes, which could potentially limit the comprehensiveness and applicability of our findings to typical clinical scenarios.

Here are the highlighted modifications in the manuscript:

**Revised Introduction Text:**

"Given the critical importance of identifying and addressing psychological barriers to successful rehabilitation, a comprehensive synthesis of available evidence from observational studies is warranted. Such an endeavor would not only clarify the extent to which depression and anxiety disorders influence THA and TKA outcomes but also identify potential areas where targeted interventions could be most beneficial. Observational studies, by capturing data from routine clinical settings, offer direct insights into the real-world impacts of these psychological conditions on surgical outcomes.”

(Page 8)

**Revised Discussion Text:**

"Furthermore, while this meta-analysis provides comprehensive insights from observational studies, it is important to acknowledge that only observational studies were included. The exclusion of randomized controlled trials, due to their limited availability on the subject, may affect the generalizability of the findings. Observational studies can include biases not typically present in randomized controlled trials, which should be considered when interpreting the results.”

(Page 18)
