# Supplementary material for: The impact of depression and anxiety disorders on postoperative outcomes for patients having total hip or knee arthroplasty: Protocol of a meta-analytic study from cohort studies
Source: PLoS One. 2025 Jan 27;20(1):e0318067. doi: 10.1371/journal.pone.0318067 (PMC11771941; doi:10.1371/journal.pone.0318067)
Supplement: Peer review history

# Editor report: Decision Letter 0

**DOI:** 10.1371/journal.pone.0318067.r001

**Authors:** Sultana Monira Hussain

15 Dec 2024

PONE-D-24-55368The impact of depression and anxiety disorders on postoperative outcomes for patients having total hip or knee arthroplasty: protocol of a meta-analytic study from cohort studiesPLOS ONE

Dear Dr. Mei,

Thank you for submitting your manuscript to PLOS ONE. After careful consideration, we feel that it has merit but does not fully meet PLOS ONE’s publication criteria as it currently stands. Therefore, we invite you to submit a revised version of the manuscript that addresses the points raised during the review process.

Please submit your revised manuscript by Jan 29 2025 11:59PM. If you will need more time than this to complete your revisions, please reply to this message or contact the journal office at plosone@plos.org. When you're ready to submit your revision, log on to https://www.editorialmanager.com/pone/ and select the 'Submissions Needing Revision' folder to locate your manuscript file.

Please include the following items when submitting your revised manuscript:

- A rebuttal letter that responds to each point raised by the academic editor and reviewer(s). You should upload this letter as a separate file labeled 'Response to Reviewers'.
- A marked-up copy of your manuscript that highlights changes made to the original version. You should upload this as a separate file labeled 'Revised Manuscript with Track Changes'.
- An unmarked version of your revised paper without tracked changes. You should upload this as a separate file labeled 'Manuscript'.

If you would like to make changes to your financial disclosure, please include your updated statement in your cover letter. Guidelines for resubmitting your figure files are available below the reviewer comments at the end of this letter.

If applicable, we recommend that you deposit your laboratory protocols in protocols.io to enhance the reproducibility of your results. Protocols.io assigns your protocol its own identifier (DOI) so that it can be cited independently in the future. For instructions see: https://journals.plos.org/plosone/s/submission-guidelines#loc-laboratory-protocols. Additionally, PLOS ONE offers an option for publishing peer-reviewed Lab Protocol articles, which describe protocols hosted on protocols.io. Read more information on sharing protocols at https://plos.org/protocols?utm_medium=editorial-email&utm_source=authorletters&utm_campaign=protocols.

We look forward to receiving your revised manuscript.

Kind regards,

Sultana Monira Hussain

Academic Editor

PLOS ONE

Journal Requirements:

When submitting your revision, we need you to address these additional requirements.

1. Please ensure that your manuscript meets PLOS ONE's style requirements, including those for file naming. The PLOS ONE style templates can be found at 

https://journals.plos.org/plosone/s/file?id=wjVg/PLOSOne_formatting_sample_main_body.pdf and 

https://journals.plos.org/plosone/s/file?id=ba62/PLOSOne_formatting_sample_title_authors_affiliations.pdf

2. Your ethics statement should only appear in the Methods section of your manuscript. If your ethics statement is written in any section besides the Methods, please move it to the Methods section and delete it from any other section. Please ensure that your ethics statement is included in your manuscript, as the ethics statement entered into the online submission form will not be published alongside your manuscript.

Additional Editor Comments:

Dear Authors,

Thank you for your submission. I have a few suggestions that I believe will enhance the rigor and depth of your study:

Bias Assessment: I recommend using a more robust tool for bias assessment, such as the ROBINS-I or JBI tools, rather than the Newcastle-Ottawa Scale. The latter relies on a points system that may not adequately reflect the complexity of bias in observational studies.

Exposure Assessment: For evaluating exposures such as depression and anxiety, I suggest incorporating PsycINFO into your database search strategy. This database specializes in psychological literature and may provide additional relevant studies.

Subgroup Analysis: In addition to the proposed subgroup analyses, I suggest including a subgroup analysis based on the severity of pain prior to arthroplasty. This variable may influence both psychological factors and postoperative outcomes.

Inclusion of RCTs: If available, I recommend incorporating randomized controlled trials (RCTs) that specifically address the management of psychological disorders (e.g., depression, anxiety) before performing total knee arthroplasty (TKA) or total hip arthroplasty (THA).

[Note: HTML markup is below. Please do not edit.]

[NOTE: If reviewer comments were submitted as an attachment file, they will be attached to this email and accessible via the submission site. Please log into your account, locate the manuscript record, and check for the action link "View Attachments". If this link does not appear, there are no attachment files.]

While revising your submission, please upload your figure files to the Preflight Analysis and Conversion Engine (PACE) digital diagnostic tool, https://pacev2.apexcovantage.com/. PACE helps ensure that figures meet PLOS requirements. To use PACE, you must first register as a user. Registration is free. Then, login and navigate to the UPLOAD tab, where you will find detailed instructions on how to use the tool. If you encounter any issues or have any questions when using PACE, please email PLOS at figures@plos.org. Please note that Supporting Information files do not need this step.

# Author comment: Author response to Decision Letter 0

**DOI:** 10.1371/journal.pone.0318067.r002

21 Dec 2024

Detailed Rebuttal Letter to Editor and Reviewer

Ms. No.: PONE-D-24-55368

To: The impact of depression and anxiety disorders on postoperative outcomes for patients having total hip or knee arthroplasty: protocol of a meta-analytic study from cohort studies

Prof. Sultana Monira Hussain, 

Academic Editor

PLOS ONE

Dear Prof. Sultana Monira Hussain, 

Thank you for forwarding the review comments to us. We have carefully gone through the feedback and have made the necessary changes to our manuscript. We appreciate the insightful feedback provided by the editor and reviewers, as they have helped us improve the quality of our study. Please find below a detailed list of the changes made in response to the Editor and the Reviewer's concerns:

Editor’s comments

Thank you for submitting your manuscript to PLOS ONE. After careful consideration, we feel that it has merit but does not fully meet PLOS ONE’s publication criteria as it currently stands. Therefore, we invite you to submit a revised version of the manuscript that addresses the points raised during the review process.

Please submit your revised manuscript by Jan 29 2025 11:59PM. If you will need more time than this to complete your revisions, please reply to this message or contact the journal office at plosone@plos.org. When you're ready to submit your revision, log on to https://www.editorialmanager.com/pone/ and select the 'Submissions Needing Revision' folder to locate your manuscript file.

Please include the following items when submitting your revised manuscript:

A rebuttal letter that responds to each point raised by the academic editor and reviewer(s). You should upload this letter as a separate file labeled 'Response to Reviewers'.

A marked-up copy of your manuscript that highlights changes made to the original version. You should upload this as a separate file labeled 'Revised Manuscript with Track Changes'.

An unmarked version of your revised paper without tracked changes. You should upload this as a separate file labeled 'Manuscript'.

If you would like to make changes to your financial disclosure, please include your updated statement in your cover letter. Guidelines for resubmitting your figure files are available below the reviewer comments at the end of this letter.

If applicable, we recommend that you deposit your laboratory protocols in protocols.io to enhance the reproducibility of your results. Protocols.io assigns your protocol its own identifier (DOI) so that it can be cited independently in the future. For instructions see: https://journals.plos.org/plosone/s/submission-guidelines#loc-laboratory-protocols. Additionally, PLOS ONE offers an option for publishing peer-reviewed Lab Protocol articles, which describe protocols hosted on protocols.io. Read more information on sharing protocols at https://plos.org/protocols?utm_medium=editorial-email&utm_source=authorletters&utm_campaign=protocols.

We look forward to receiving your revised manuscript.

Kind regards,

Sultana Monira Hussain

Academic Editor

PLOS ONE.

Response: Thank you for your consideration of our manuscript. We appreciate the opportunity to revise our manuscript to meet PLOS ONE’s publication criteria and address the review comments comprehensively.

We acknowledge the deadline of January 29, 2025, for the submission of the revised manuscript. We anticipate that we will be able to complete the necessary revisions by this date. However, should we require additional time, we will notify you via plosone@plos.org.

In our revised submission, we will include the following documents as instructed:

1. A rebuttal letter addressing each point raised by the academic editor and reviewers, detailing the changes made in response to their feedback.

2. A marked-up copy of the manuscript highlighting the revisions.

3. A clean version of the manuscript without tracked changes for ease of reading.

4. An updated financial disclosure in the cover letter, if applicable.

We also appreciate the suggestion to deposit our laboratory protocols in protocols.io to enhance reproducibility. As this is a study protocol, all the methods have been described in details in the full manuscript.

Thank you for the opportunity to improve our manuscript. We look forward to the possibility of contributing to PLOS ONE and will ensure our revised submission fulfills the journal’s standards for publication.

Journal Requirements:

When submitting your revision, we need you to address these additional requirements.

1. Please ensure that your manuscript meets PLOS ONE's style requirements, including those for file naming. The PLOS ONE style templates can be found at 

https://journals.plos.org/plosone/s/file?id=wjVg/PLOSOne_formatting_sample_main_body.pdf and 

https://journals.plos.org/plosone/s/file?id=ba62/PLOSOne_formatting_sample_title_authors_affiliations.pdf.

Response: Thank you for the guidance regarding the PLOS ONE style requirements. We acknowledge the importance of adhering to the journal's formatting standards and have made the necessary revisions to comply with these guidelines.

Using the PLOS ONE templates provided in the links, we have reformatted our manuscript to match the required style. This includes adjustments to the title page, headings, subheadings, font sizes, and the overall layout to ensure compliance with the journal’s formatting guidelines.

We hope these revisions meet the journal's requirements. Attached are the revised files for your review and consideration. We appreciate the opportunity to refine our manuscript and look forward to the possibility of its acceptance in PLOS ONE.

2. Your ethics statement should only appear in the Methods section of your manuscript. If your ethics statement is written in any section besides the Methods, please move it to the Methods section and delete it from any other section. Please ensure that your ethics statement is included in your manuscript, as the ethics statement entered into the online submission form will not be published alongside your manuscript.

Response: Thank you for your guidance regarding the placement of the ethics statement in our manuscript. We understand the importance of correctly positioning this statement to adhere to the journal's standards.

We have carefully reviewed our manuscript and have made the following adjustments:

1. We have moved the ethics statement exclusively to the Methods section of our manuscript. This adjustment ensures that all ethical disclosures are appropriately centralized and easily accessible, aligning with best practices for transparency and reproducibility.

2. We have removed the ethics statement from Abstract section of the manuscript where it previously appeared, to prevent redundancy and maintain a clean and focused presentation of our research.

3. We have ensured that the ethics statement is included in the manuscript as per your instructions. This will guarantee that the statement is available in the published article, as the ethics information provided during the online submission process is not published alongside the manuscript.

We appreciate your attention to the ethical aspects of publication and adherence to PLOS ONE’s policies. We hope these revisions satisfactorily address your comments regarding the ethics statement.

Additional Editor Comments:

Dear Authors,

Thank you for your submission. I have a few suggestions that I believe will enhance the rigor and depth of your study:

Bias Assessment: I recommend using a more robust tool for bias assessment, such as the ROBINS-I or JBI tools, rather than the Newcastle-Ottawa Scale. The latter relies on a points system that may not adequately reflect the complexity of bias in observational studies.

Response: Thank you for your valuable comments and suggestions aimed at enhancing the rigor of our study. We appreciate your recommendation regarding the bias assessment tool and agree that a more robust method would strengthen our meta-analysis. We acknowledge the limitations of the Newcastle-Ottawa Scale (NOS) in reflecting the complexity of bias in observational studies. In response to your suggestion, we have decided to adopt the ROBINS-I tool for bias assessment, which is specifically designed for reviewing non-randomized studies of interventions. This tool will allow us to more comprehensively assess biases due to confounding, selection of participants, classification of interventions, deviations from intended interventions, missing data, measurement of outcomes, and selection of reported results.

Revised Method Text:

"Risk of Bias Assessment

To ensure the reliability and validity of the synthesized evidence, a rigorous risk of bias assessment will be conducted for all included studies. For non-randomized cohort studies, the ROBINS-I tool will be applied to evaluate seven domains of bias: confounding, selection of participants into the study, classification of interventions, deviations from intended interventions, missing data, measurement of outcomes, and selection of reported results [37]. Judgments for each domain will inform an overall risk of bias assessment for each study, categorized as 'low', 'moderate', 'serious', or 'critical'. Disagreements in the risk of bias assessment will also be resolved through discussion among reviewers or consultation with a third expert reviewer when necessary.

(Page 13)

Exposure Assessment: For evaluating exposures such as depression and anxiety, I suggest incorporating PsycINFO into your database search strategy. This database specializes in psychological literature and may provide additional relevant studies.

Response: Thank you for your constructive suggestion regarding the enhancement of our search strategy. We agree that incorporating PsycINFO is a valuable addition that could significantly enrich our review by providing comprehensive coverage of psychological literature, which is crucial for our research focus on depression and anxiety. We recognize the importance of thoroughly capturing studies related to the psychological aspects of orthopedic postoperative outcomes. PsycINFO, with its specialized focus, will likely yield additional relevant studies that may not be indexed in the databases previously included in our search strategy.

Revised Abstract Text:

“Methods and Analysis: 

We will search PubMed, Embase, Cochrane Library and PsycINFO from inception to the November 2024, adopting a comprehensive search strategy with no language restrictions.”

(Page 4)

Revised Method Text:

"Search Strategy

A systematic search of electronic databases, including PubMed, Embase, the Cochrane Library, and PsycINFO will be conducted from their inception to November 2024. Search terms will include a combination of keywords and Medical Subject Headings (MeSH) related to "total hip arthroplasty," "total knee arthroplasty," "depression," "anxiety disorders," "postoperative outcomes," and "cohort studies." The search strategy will be adapted for each database, and no language restrictions will initially be applied to maximize sensitivity (details see Table 1-3). Reference lists of included studies and relevant reviews will be manually screened to identify additional eligible studies.”

(Page 11-12)

Subgroup Analysis: In addition to the proposed subgroup analyses, I suggest including a subgroup analysis based on the severity of pain prior to arthroplasty. This variable may influence both psychological factors and postoperative outcomes.

Response: Thank you for your insightful comment regarding the incorporation of pain severity in our subgroup analyses. We agree that the severity of preoperative pain is a critical factor that could influence both the psychological state of patients and their outcomes after total hip or knee arthroplasty.

Including a subgroup analysis based on the severity of preoperative pain will allow us to more deeply understand how pain interacts with depression and anxiety to affect postoperative results. Pain severity can significantly affect patient psychology, recovery, and satisfaction with surgical outcomes, thus it is indeed a pertinent variable to include in our analyses.

Revised Method Text:

" Subgroup analyses will be conducted based on factors such as age group (adult vs. elderly), type of arthroplasty (THA vs. TKA), severity of depression/anxiety, severity of preoperative pain, and study quality, to explore potential sources of heterogeneity."

(Page 15)

Inclusion of RCTs: If available, I recommend incorporating randomized controlled trials (RCTs) that specifically address the management of psychological disorders (e.g., depression, anxiety) before performing total knee arthroplasty (TKA) or total hip arthroplasty (THA).

Response: Thank you for the reviewer's suggestion to include randomized controlled trials (RCTs) that address the management of psychological disorders before total knee arthroplasty (TKA) or total hip arthroplasty (THA). We appreciate the potential value that such studies might offer in understanding the impact of treatment for psychological disorders on surgical outcomes.

However, after careful consideration and a thorough search of the available literature, we have found that RCTs specifically examining the impact of treating depression and anxiety before TKA and THA are exceedingly scarce. The primary reason for this lack of RCTs is the ethical and logistical challenges associated with randomly assigning patients to different treatment modalities for psychological conditions in the context of preparing for major surgery. Such interventions typically require personalized treatment plans that take into account the severity and specifics of the individual's psychological condition, which do not lend themselves well to randomization.

Moreover, the inclusion of RCTs in our study would potentially introduce a significant methodological shift from our original design, which is focused on observational cohort studies. Cohort studies are particularly suitable for our research question as they allow the examination of real-world outcomes in routine clinical practice, providing insights into the naturalistic effects of depression and anxiety on surgical results.

Additionally, the heterogeneity in intervention types, variability in the timing of interventions relative to surgical dates, and differences in outcome measures reported in RCTs compared to observational studies would complicate the synthesis of data and could lead to misleading conclusions.

Therefore, we have chosen to focus our meta-analysis on cohort studies, which provide a more homogeneous and directly applicable body of evidence to assess the impact of pre-existing depression and anxiety on postoperative outcomes in TKA and THA patients.

Attachment Submitted filename: Response to Reviewers.docx

# Editor report: Decision Letter 1

**DOI:** 10.1371/journal.pone.0318067.r003

**Authors:** Sultana Monira Hussain

6 Jan 2025

PONE-D-24-55368R1The impact of depression and anxiety disorders on postoperative outcomes for patients having total hip or knee arthroplasty: protocol of a meta-analytic study from cohort studiesPLOS ONE

Dear Dr. Mei,

Thank you for submitting your manuscript to PLOS ONE. After careful consideration, we feel that it has merit but does not fully meet PLOS ONE’s publication criteria as it currently stands. Therefore, we invite you to submit a revised version of the manuscript that addresses the points raised during the review process.

Please submit your revised manuscript by Feb 20 2025 11:59PM. If you will need more time than this to complete your revisions, please reply to this message or contact the journal office at plosone@plos.org. When you're ready to submit your revision, log on to https://www.editorialmanager.com/pone/ and select the 'Submissions Needing Revision' folder to locate your manuscript file.

Please include the following items when submitting your revised manuscript:

- A rebuttal letter that responds to each point raised by the academic editor and reviewer(s). You should upload this letter as a separate file labeled 'Response to Reviewers'.
- A marked-up copy of your manuscript that highlights changes made to the original version. You should upload this as a separate file labeled 'Revised Manuscript with Track Changes'.
- An unmarked version of your revised paper without tracked changes. You should upload this as a separate file labeled 'Manuscript'.

If you would like to make changes to your financial disclosure, please include your updated statement in your cover letter. Guidelines for resubmitting your figure files are available below the reviewer comments at the end of this letter.

If applicable, we recommend that you deposit your laboratory protocols in protocols.io to enhance the reproducibility of your results. Protocols.io assigns your protocol its own identifier (DOI) so that it can be cited independently in the future. For instructions see: https://journals.plos.org/plosone/s/submission-guidelines#loc-laboratory-protocols. Additionally, PLOS ONE offers an option for publishing peer-reviewed Lab Protocol articles, which describe protocols hosted on protocols.io. Read more information on sharing protocols at https://plos.org/protocols?utm_medium=editorial-email&utm_source=authorletters&utm_campaign=protocols.

We look forward to receiving your revised manuscript.

Kind regards,

Sultana Monira Hussain

Academic Editor

PLOS ONE

**Journal Requirements:**

Please review your reference list to ensure that it is complete and correct. If you have cited papers that have been retracted, please include the rationale for doing so in the manuscript text, or remove these references and replace them with relevant current references. Any changes to the reference list should be mentioned in the rebuttal letter that accompanies your revised manuscript. If you need to cite a retracted article, indicate the article’s retracted status in the References list and also include a citation and full reference for the retraction notice.

**Additional Editor Comments:**

Thank you for your detailed response and the thorough explanation regarding the inclusion of randomized controlled trials (RCTs). I appreciate the thoughtful rationale you have provided. However, to ensure the clarity and robustness of your manuscript, I recommend addressing the following points in your revision:

At the beginning of the manuscript, please clearly articulate why observational studies were chosen as the primary focus. Highlight their relevance and applicability to your research question. This will help set the stage for readers and provide a clear framework for understanding your approach. Additionally, explicitly acknowledge in the limitations section that you have only included observational studies.

Alternatively, if you decide to include both observational studies and RCTs, please acknowledge the limitations, noting the scarcity of RCTs in this area. Include a brief summary of how many relevant RCTs were identified during your literature search (if any) and the reasons they could not be incorporated. Furthermore, if there are heterogeneities between observational studies and RCTs in your meta-analysis, I suggest conducting a subgroup analysis to account for potential differences.

Addressing these points will enhance the transparency and scientific rigor of your work. I look forward to reviewing your revised manuscript.

Best regards,

Sultana Monira Hussain

[Note: HTML markup is below. Please do not edit.]

[NOTE: If reviewer comments were submitted as an attachment file, they will be attached to this email and accessible via the submission site. Please log into your account, locate the manuscript record, and check for the action link "View Attachments". If this link does not appear, there are no attachment files.]

While revising your submission, please upload your figure files to the Preflight Analysis and Conversion Engine (PACE) digital diagnostic tool, https://pacev2.apexcovantage.com/. PACE helps ensure that figures meet PLOS requirements. To use PACE, you must first register as a user. Registration is free. Then, login and navigate to the UPLOAD tab, where you will find detailed instructions on how to use the tool. If you encounter any issues or have any questions when using PACE, please email PLOS at figures@plos.org. Please note that Supporting Information files do not need this step.

# Author comment: Author response to Decision Letter 1

**DOI:** 10.1371/journal.pone.0318067.r004

6 Jan 2025

Detailed Rebuttal Letter to Editor and Reviewer

Ms. No.: PONE-D-24-55368R1

To: The impact of depression and anxiety disorders on postoperative outcomes for patients having total hip or knee arthroplasty: protocol of a meta-analytic study from cohort studies

Prof. Sultana Monira Hussain, 

Academic Editor

PLOS ONE

Dear Prof. Sultana Monira Hussain, 

Thank you for forwarding the review comments to us. We have carefully gone through the feedback and have made the necessary changes to our manuscript. We appreciate the insightful feedback provided by the editor and reviewers, as they have helped us improve the quality of our study. Please find below a detailed list of the changes made in response to the Editor and the Reviewer's concerns:

Editor’s comments

Thank you for submitting your manuscript to PLOS ONE. After careful consideration, we feel that it has merit but does not fully meet PLOS ONE’s publication criteria as it currently stands. Therefore, we invite you to submit a revised version of the manuscript that addresses the points raised during the review process.

Please submit your revised manuscript by Feb 20 2025 11:59PM. If you will need more time than this to complete your revisions, please reply to this message or contact the journal office at plosone@plos.org. When you're ready to submit your revision, log on to https://www.editorialmanager.com/pone/ and select the 'Submissions Needing Revision' folder to locate your manuscript file.

Please include the following items when submitting your revised manuscript:

A rebuttal letter that responds to each point raised by the academic editor and reviewer(s). You should upload this letter as a separate file labeled 'Response to Reviewers'.

A marked-up copy of your manuscript that highlights changes made to the original version. You should upload this as a separate file labeled 'Revised Manuscript with Track Changes'.

An unmarked version of your revised paper without tracked changes. You should upload this as a separate file labeled 'Manuscript'.

If you would like to make changes to your financial disclosure, please include your updated statement in your cover letter. Guidelines for resubmitting your figure files are available below the reviewer comments at the end of this letter.

If applicable, we recommend that you deposit your laboratory protocols in protocols.io to enhance the reproducibility of your results. Protocols.io assigns your protocol its own identifier (DOI) so that it can be cited independently in the future. For instructions see: https://journals.plos.org/plosone/s/submission-guidelines#loc-laboratory-protocols. Additionally, PLOS ONE offers an option for publishing peer-reviewed Lab Protocol articles, which describe protocols hosted on protocols.io. Read more information on sharing protocols at https://plos.org/protocols?utm_medium=editorial-email&utm_source=authorletters&utm_campaign=protocols.

We look forward to receiving your revised manuscript.

Kind regards,

Sultana Monira Hussain

Academic Editor

PLOS ONE.

Response: Thank you for your consideration of our manuscript. We appreciate the opportunity to revise our manuscript to meet PLOS ONE’s publication criteria and address the review comments comprehensively.

We acknowledge the deadline of January 29, 2025, for the submission of the revised manuscript. We anticipate that we will be able to complete the necessary revisions by this date. However, should we require additional time, we will notify you via plosone@plos.org.

In our revised submission, we will include the following documents as instructed:

1.A rebuttal letter addressing each point raised by the academic editor and reviewers, detailing the changes made in response to their feedback.

2.A marked-up copy of the manuscript highlighting the revisions.

3.A clean version of the manuscript without tracked changes for ease of reading.

4.An updated financial disclosure in the cover letter, if applicable.

We also appreciate the suggestion to deposit our laboratory protocols in protocols.io to enhance reproducibility. As this is a study protocol, all the methods have been described in details in the full manuscript.

Thank you for the opportunity to improve our manuscript. We look forward to the possibility of contributing to PLOS ONE and will ensure our revised submission fulfills the journal’s standards for publication.

Journal Requirements:

Please review your reference list to ensure that it is complete and correct. If you have cited papers that have been retracted, please include the rationale for doing so in the manuscript text, or remove these references and replace them with relevant current references. Any changes to the reference list should be mentioned in the rebuttal letter that accompanies your revised manuscript. If you need to cite a retracted article, indicate the article’s retracted status in the References list and also include a citation and full reference for the retraction notice.

Response: Thank you for the feedback. We have reviewed our reference list thoroughly to ensure it is complete, accurate, and up-to-date. None of the articles cited in our manuscript have been retracted. We have confirmed this by checking each citation against databases like Retraction Watch and PubMed. Furthermore, all references are now double-checked for accuracy and completeness, adhering to the journal's citation style.

Additional Editor Comments:

Thank you for your detailed response and the thorough explanation regarding the inclusion of randomized controlled trials (RCTs). I appreciate the thoughtful rationale you have provided. However, to ensure the clarity and robustness of your manuscript, I recommend addressing the following points in your revision:

At the beginning of the manuscript, please clearly articulate why observational studies were chosen as the primary focus. Highlight their relevance and applicability to your research question. This will help set the stage for readers and provide a clear framework for understanding your approach. Additionally, explicitly acknowledge in the limitations section that you have only included observational studies.

Alternatively, if you decide to include both observational studies and RCTs, please acknowledge the limitations, noting the scarcity of RCTs in this area. Include a brief summary of how many relevant RCTs were identified during your literature search (if any) and the reasons they could not be incorporated. Furthermore, if there are heterogeneities between observational studies and RCTs in your meta-analysis, I suggest conducting a subgroup analysis to account for potential differences.

Addressing these points will enhance the transparency and scientific rigor of your work. I look forward to reviewing your revised manuscript.

Best regards,

Sultana Monira Hussain.

Response: Thank you for your detailed comments and suggestions regarding our manuscript. In response to your feedback, we have opted to focus exclusively on observational studies for our meta-analysis. We have made the following revisions to clarify this choice in the manuscript:

In the introduction, we have articulated the rationale for choosing observational studies as our primary focus. This choice was driven by the direct applicability and real-world relevance of these studies to our research question, which investigates the impacts of depression and anxiety on postoperative outcomes in total hip and knee arthroplasty. Observational studies provide valuable insights into patient populations under routine clinical conditions, which is critical for understanding the nuances of how psychological factors affect surgical outcomes.

Furthermore, we have acknowledged in the limitations section that our analysis is based solely on observational studies. This is an important consideration for interpreting the results, as these study designs inherently carry a risk of bias that is typically lower in randomized controlled trials (RCTs). We decided against including RCTs due to their scarcity in examining the psychological aspects of orthopedic outcomes, which could potentially limit the comprehensiveness and applicability of our findings to typical clinical scenarios.

Here are the highlighted modifications in the manuscript:

Revised Introduction Text:

"Given the critical importance of identifying and addressing psychological barriers to successful rehabilitation, a comprehensive synthesis of available evidence from observational studies is warranted. Such an endeavor would not only clarify the extent to which depression and anxiety disorders influence THA and TKA outcomes but also identify potential areas where targeted interventions could be most beneficial. Observational studies, by capturing data from routine clinical settings, offer direct insights into the real-world impacts of these psychological conditions on surgical outcomes.”

(Page 8)

Revised Discussion Text:

"Furthermore, while this meta-analysis provides comprehensive insights from observational studies, it is important to acknowledge that only observational studies were included. The exclusion of randomized controlled trials, due to their limited availability on the subject, may affect the generalizability of the findings. Observational studies can include biases not typically present in randomized controlled trials, which should be considered when interpreting the results.”

(Page 18)

Attachment Submitted filename: Response to Reviewers.docx

# Editor report: Decision Letter 2

**DOI:** 10.1371/journal.pone.0318067.r005

**Authors:** Sultana Monira Hussain

10 Jan 2025

The impact of depression and anxiety disorders on postoperative outcomes for patients having total hip or knee arthroplasty: protocol of a meta-analytic study from cohort studies

PONE-D-24-55368R2

Dear Dr. Mei,

We’re pleased to inform you that your manuscript has been judged scientifically suitable for publication and will be formally accepted for publication once it meets all outstanding technical requirements.

Within one week, you’ll receive an e-mail detailing the required amendments. When these have been addressed, you’ll receive a formal acceptance letter and your manuscript will be scheduled for publication.

An invoice will be generated when your article is formally accepted. Please note, if your institution has a publishing partnership with PLOS and your article meets the relevant criteria, all or part of your publication costs will be covered. Please make sure your user information is up-to-date by logging into Editorial Manager at Editorial Manager® and clicking the ‘Update My Information' link at the top of the page. If you have any questions relating to publication charges, please contact our Author Billing department directly at authorbilling@plos.org.

If your institution or institutions have a press office, please notify them about your upcoming paper to help maximize its impact. If they’ll be preparing press materials, please inform our press team as soon as possible -- no later than 48 hours after receiving the formal acceptance. Your manuscript will remain under strict press embargo until 2 pm Eastern Time on the date of publication. For more information, please contact onepress@plos.org.

Kind regards,

Sultana Monira Hussain

Academic Editor

PLOS ONE

# Editor report: Acceptance letter

**DOI:** 10.1371/journal.pone.0318067.r006

**Authors:** Sultana Monira Hussain

14 Jan 2025

PONE-D-24-55368R2 

PLOS ONE

Dear Dr. Mei, 

I'm pleased to inform you that your manuscript has been deemed suitable for publication in PLOS ONE. Congratulations! Your manuscript is now being handed over to our production team.

At this stage, our production department will prepare your paper for publication. This includes ensuring the following:

* All references, tables, and figures are properly cited

* All relevant supporting information is included in the manuscript submission,

* There are no issues that prevent the paper from being properly typeset

If revisions are needed, the production department will contact you directly to resolve them. If no revisions are needed, you will receive an email when the publication date has been set. At this time, we do not offer pre-publication proofs to authors during production of the accepted work. Please keep in mind that we are working through a large volume of accepted articles, so please give us a few weeks to review your paper and let you know the next and final steps. 

Lastly, if your institution or institutions have a press office, please let them know about your upcoming paper now to help maximize its impact. If they'll be preparing press materials, please inform our press team within the next 48 hours. Your manuscript will remain under strict press embargo until 2 pm Eastern Time on the date of publication. For more information, please contact onepress@plos.org.

If we can help with anything else, please email us at customercare@plos.org.

Thank you for submitting your work to PLOS ONE and supporting open access. 

Kind regards, 

PLOS ONE Editorial Office Staff

on behalf of

Dr. Sultana Monira Hussain 

Academic Editor

PLOS ONE
